# A Study of the Relationship between the Eruption of the Permanent Teeth and the Ailments of Late Childhood

**Published:** 1890-06

**Authors:** Louis Starr

**Affiliations:** Philadelphia, Pa.


### SELECTIONS.

### A Study of the Relationship between the Eruption of the Permanent Teeth and the Ailments of Late Childhood.

By Louis Starr, M.D., Philadelphia, Pa.

### ERUPTION OF THE PERMANENT TEETH.

The eruption of the milk teeth, though physiological, is so uniformly regarded as dangerous, that both physicians and parents congratulate themselves when infants under their charge pass through this process without trouble, or safely weather the various diseas s that may arise during its course. The interval between the fourth and thirtieth months of an infant's life—the period of primary dentition—is an era of great and widely-extended physical progress. The teeth are advancing; the follicular apparatus of the stomach and intestinal canal is undergoing development in preparation for the digestion and absorption of mixed food; the cerebro-spinal system is rapidly growing and functionally very active, and the organs and tissues of the whole body are in a state of active change. This period of normal transition must also be one in which there is great susceptibility to abnormal change or disease, provided there be a causal influence at work. Such an influence may either originate outside of the body, as when there is exposure to cold or to contagion, or come from within in the form of some perversion of a physiological process.

Difficult dentition stands prominent in the latter class. During it, irritation of the gums very often produces stomatitis with fever, and the fever in turn leads to enfeebled digestion and impaired nutrition, conditions that open the way to catarrh of the mucous membrane of the bronchial tubes and gastro-intestinal tract, and to other intercurrent affections. Again, the local irritation may be reflected through the widely extended connection of the dental nerves, and give rise to well-known disorders of distant organs and tissues, for example, the brain, eyes, stomach, skin, and so on.

With these conditions fully recognized it is surprising that second dentition has not been accorded the position it deserves as a cause of ill-health in later childhood. In second dentition the elements of local irritation with fever are quite as potent as before. There is, however, less activity of development or rapidity of change, if we except the radical alteration in the system occasioned by the approach of puberty. Therefore there must be less susceptibility to disease, and this fact, taken with the greater resisting power of advancing age, fully accounts for what every observer will find to be true—namely, that the disorders of this period are less frequent, and, as a rule, less dangerous than those that attend the cutting of the milk teeth. Nevertheless, the etiological relations of the eruption of the permanent teeth will fully repay a careful study.

This subject will be considered here in its relation to the time between the fifth year and the establishment of puberty, when childhood is over and youth or maidenhood begins.

The permanent teeth are cut in the following order:

1. Four first molars, five to six years.
2. Four central incisors, six to eight years.
3. Four lateral incisors, seven to nine years.
4. Four first bicuspids, nine to ten years.
5. Four second bicuspids, ten to twelve years.
6. Four canines, eleven to thirteen years.
7. Four second molars, twelve to fourteen years.
8. Four posterior molars (or "wisdom teeth," not entering into this study), seventeen to twenty-one years.

Of the twenty-eight teeth cut within the period already mentioned—the fifth to the fifteenth years—the first and seventh sets are developed *de novo*, and are more likely to give rise to trouble, particularly to the oral mucous membrane. The other sets take the place of corresponding milk teeth, and appear in very much the same order, the lower central incisors appearing before the upper, the upper lateral incisors before the lower, the upper bicuspids before the lower.

As these teeth approach the surface absorption begins in the alveoli and at the roots of the deciduous teeth, and this continues until the teeth are loosened and readily extracted, or, if this be not done, until little is left but their crowns.

When the first and second molars approach the surface, the gums, just as in primary dentition, become red, swollen, rounded and tender. The salivary secretion is increased, the mouth is hot, the patient complains of aching in the gum, and, on account of tenderness, refuses food requiring mastication. With the other sets there is a gradual loosening of the super-imposed temporary teeth, pain on mastication, redness and tumefaction of the gum, and augmented flow of saliva. As there is no impairment of the general health, these trifling symptoms must be regarded merely as manifestations of the progress of a physiological process. Such are the normal manifestations.

The most common disorders of second dentition are (*a*) those of the mouth and throat; (*b*) of general nutrition; (*c*) of the stomach and intestinal canal; (*d*) of the cervical lymphatic glands; (*e*) of the eyes; (*f*) of the ears; (*g*) of the skin; (*h*) of the respiratory tract; and (*i*) of the nervous system.

*a.* Oral pain is often intense. It is lancinating or neuralgic in character, and may either be limited to the position of the advancing tooth or extend throughout the upper and lower jaws—the region supplied by the dental branches of the trifacial nerve. Sometimes the pain is referred to the eye, the ear, the face, or even to the forehead. Pain associated with tenderness most frequently attends the eruption of the first molars; then there is also redness and marked swelling of the gum, as in primary dentition.

The redness and swelling about an advancing tooth, or around a loosened milk tooth, may, in debilitated or strumous subjects, extend to the mucous membrane of the whole mouth and give rise to catarrhal stomatitis. Again, as one of the first or second molars advances, the mucous membrane of the gum, directly over the tooth, breaks down and a circular ulcer is formed. This ulcer possesses all the characteristics of the marginal ulcer of ulcerative stomatitis, and is very liable—provided such favoring conditions as scrofula and bad hygiene exist—to run around the alveolar border and extend to the inside of the cheek. A case has recently been under my care in which all of the six-year molars were cut in this way, the resulting ulcerative stomatitis producing considerable discomfort, but yielding readily to treatment.

Superficial ulcers upon the edges and tip of the tongue are often encountered. These ulcers correspond in position and number to loosened and, perhaps, decaying deciduous teeth; are due to constant irritation of the mucous membrane; are the seat of moderate pain, and more or less interfere with the movements of the organ in mastication and speech. They vary in shape and size, but are generally oval, with the greater diameter—rarely more than half an inch—extending in the direction of the axis of the tongue. Their bases are smooth, red, and shining, and their edges red, indented, somewhat indurated, and surrounded by a narrow band of white fur formed upon the neighboring healthy epithelium.

A boy, 10 years old, was recently brought to consult me about the condition of his tongue. Nearly two months before the anterior deciduous molars had commenced to loosen. Soon after two ulcers appeared upon the tongue at points corresponding to the loose teeth in the lower jaw. These caused considerable discomfort and interfered with the movements of his tongue. Six weeks later the four loose teeth were extracted. At the time of his visit the points of the permanent teeth were distinctly visible. The ulcers, which presented the characteristics already described, were present, too, but they were much contracted and evidently in process of healing. This case is a clear illustration of the etiology of the condition, and of the rapid effect of the removal of the cause.

Loss or perversion of taste, depending upon reflected irritation of the gustatory and glosso-pharyngeal nerves, has occasionally arisen in my experience. It is a feature that may be readily overlooked in childhood, and without doubt, has never received due credit as a cause of the anorexia so often observed during second dentition.

Of throat affections, simple hypertrophy of the tonsils and follicular tonsillitis seem particularly apt to arise in late child hood. The extension of catarrhal inflammation from the mouth to the throat is certainly an element in the causation of the conditions, though anorexia, imperfect digestion, and fever are more potent, as they lead to impaired nutrition and increased susceptibillty to the action of cold and bad hygienic surroundings.

The treatment of this class of affections must vary with the symptoms presented. Should there be much inflammtion and pain about a loose tooth great relief can be obtained by painting the gum three or four times daily with a solution of—

℞. Cocain. hydrochlorat..........................gr. iv.

Glycerinæ....................................fʒ ii.

Aquæ q. s., ad..............................f℥ i.

M. Sig. For local use.

When the first or second molars cause the trouble free lancing with an oblique crucial incision is to be recommended. Much good can also be done in the way of softening the gums and lessening pain by a thorough application of—

℞. Zinci chloridi..............................g. i.

Vin. opii....................................fʒ i.

Glycerinæ....................................fʒ ii.

Aquæ rosæ q. s., ad.........................f℥ i.

M. Sig. Apply to tender gums with a brush or soft cloth thrice daily.

Such measures, too, will be more successful in relieving referred pains than any direct application to the place of reference.

Catarrhal and ulcerative stomatitis demand the usual methods of treatment.

Superficial ulcers on the tongue can often be healed by a daily application of a solution of nitrate of silver (ten grains to one fluid-ounce), and the frequent use of a borax or chlorate of potassium wash (fifteen grains of either to one fluid-ounce). Should there be much pain and discomfort, the solution of cocaine recommended above may be used two or three times daily. When the deciduous teeth are decayed, however, nothing short of their extraction will cure the ulceration.

Nothing can be done for loss or perversion of taste except removing loose teeth and freely lancing the gum over advancing molars.

Hypertrophy of the tonsils and follicular tonsillitis must be treated in the same way as when they occur independently of dentition, the question of the propriety of extraction and of lancing being always borne in mind.

*b.* After safely passing through primary dentition, children usually grow robust and enjoy good health unless they be attacked by some one of the acute contagious diseases to which their age is liable. This state of affairs may happily endure throughout the remainder of childhood but it is often supplanted during the sixth and following years by a condition best described as one of "general debility."

This ill health is neither produced by disease of important viscera, as of the lungs, heart, kidneys, and digestive organs, nor can bad hygiene be blamed in many cases. For the explanation one must look rather to an impairment of nutrition, resulting from the constitutional strain of cutting the second teeth, from the moderate fever associated with the process, and from the diminished consumption of food attending oral discomfort and painful mastication. The severity of the symptoms depends somewhat upon the general vigor of the subject attacked, though in my experience, it bears little or no relation to the difficulty or ease of cutting the milk teeth.

Early in the sixth year children so affected begin to lose their rosy cheeks; the lips grow pale; the skin of the body becomes sallow and harsh; the hair dry and lustreless, and there is moderate loss of flesh with flabbiness of the muscles. The face wears an anxious expression, and the temper is unstable; by day frequent complaints of weariness are made and little interest is taken in play, while at night sleep is restless, and there is often slight fever with a temperature rarely above 100° F. Pain and discomfort in the mouth are constant symptoms, and as these are increased by mastication there is apparent anorexia. Examination of the mouth reveals redness, swelling and tenderness of the gums over advancing molars, or if these have been cut, around loose temporary teeth. The bowels are inclined to constipation, and the urine limpid and voided in abundant quantity; the pulse is rather feeble, though normal in frequency, and as a rule, there is no cough nor other alteration in the respiratory function. Careful investigation shows an absence of lesion in the heart, lungs, or kidneys, and of disease of the abdominal glands or digestive tract. As the teeth of the advancing group are cut, the symptoms disappear, to return with the approach of each succeeding group but the anterior molars generally give rise to more marked disturbance than any of the teeth that replace the temporary set.

In addition to the ordinary risk of intercurrent disease, existing in every case of general debility, the condition just described is very apt to be complicated by bronchitis or catarrh of the gastro-intestinal canal. Pyrexia, although it is comparatively slight in second dentition, accounts for this, for a feverish child is very susceptible to cold, and very liable to have his digestion disordered by food upon which he has previously thriven. The first cause, by driving the blood from the surface, produces bronchitis; the second, by direct and indirect irritation, leads to catarrh of the mucous membrane of the stomach and bowels.

This knowledge, taken in connection with the course and history of the case and the condition of the mouth, should enable the observer to attribute the illness to its proper source rather than to any complicating affection, although the latter undoubtedly accentuates the symptoms, and may force itself into prominence. The negative results of physical exploration of the heart, lungs, and abdominal organs—particularly the mesenteric glands—and of examination of the urine, are also important in establishing the correct relations of cause and effect.

Careful regulation of the diet and the administration of tonics, the methods of treatment that would naturally be suggested, are of little avail unless oral pain and difficulty in mastication be relieved. Even then it is often impossible to do more than maintain a moderate degree of health until the advancing teeth are completely free.

Free lancing of the gums over molars, the application of cocaine to painful gums surrounding loose temporary teeth, the extraction of these when the substituting teeth are so advanced as to run no risk of impairing the arch of the jaw, regulation of the diet and hygiene, and the employment of tonics and laxatives are the measures to be recommended. The diet must be simple, non-farinacious and nutritious; it is better to allow four small meals a day than three large ones. Of tonics, a good formula is—

| | | |
|---|---|---|
| ℞. | Tr. nucis vomicæ | ♏ xii. |
| | Elix. cinchon. ferrat | f℥ vi. |
| | Syrupi | f℥ ss. |
| | Aquæ, q. s., ad | f℥ iii. |

M. Sig. Two teaspoonfuls thrice daily at the age of six years.

Syrup of the iodide of iron, bitter wine of iron, and an emulsion of cod liver oil with lacto-phosphate of lime are also very useful. The best laxatives are fluid extract of senna, which may be combined with the tonic mixtures; tincture of aloes and myrrh in small doses three times daily, compound licorice powder, gluten suppositories, or laxative tamarinds.

Complicating bronchitis and catarrh of the gastro-intestinal canal demand active attention and little else can be accomplished until they are relieved.

*c.* Disorders of the digestive system, while unattended by such marked symptoms, and rarely reaching the same degree of danger as in primary dentition, are among the most common of the disturbances produced by the eruption of the second teeth. One cause of this is the intimate sympathy existing between the different portions of the digestive tract, and leading to reflection of irritation from point to point. Another and more active cause has already been indicated. It involves two conditions: First, general depression of vitality—from constitutional strain, fever, and so on—with a corresponding weakening of the function of digestion, so that food previously suitable and easily assimilated becomes relatively too coarse and "strong;" and being more or less imperfectly changed and absorbed, lies in the alimentary canal undergoing fermentation and decomposition with the forma tion of irritant acids and gases. Second, the well-recognized susceptibility of the gastro-intestinal mucous membrane to become inflamed, or to assume the catarrhal state when subjected to the action of irritants. A susceptibility decided enough under the best of circumstances but intensely marked if the general health and resisting power be below par.

Anorexia, vomiting, acute gastric catarrh, chronic gastro-intestinal catarrh and diarrhœa are the more common of the digestive disorders attending second dentition. Loss of appetite, when not due to perversion of taste, generally forms but one member of a group of symptoms depending on catarrh of the stomach, and can be best studied under this head. The same may be said of vomiting. Of each of these symptoms it is also true that they may be so prominent as to mask the associated features unless the observer be very careful.

Acute gastric catarrh, in so far as it is related to second dentition, is most frequently encountered during the eruption of the six-year molars. The attack of so-called "biliousness" and "indigestion," may or may not be preceded by some indiscretion in diet. Very often, without any departure from ordinary rules, the mother notices that her charge becomes listless, has a hot, dry skin, loses appetite, is thirsty, complains of headache, abdominal discomfort and nausea, and sleeps uneasily. Soon there is vomiting of sour-smelling imperfectly-digested food, followed by painful retching, with the expulsion of small quantities of bile-stained mucus. Next the tongue becomes covered with a thick, yellowish-white fur, and the breath has a heavy, sour odor. There is decided elevation of temperature (99° to 101° Fahrenheit); the pulse is increased in frequency (110 to 120); the bowels are confined and the urine diminished in quantity, and lateritious. Tenderness of the epigastrium is generally noticeable. The attack may terminate suddenly, at the end of twenty-four hours, with several loose fæcal evacuations, or convalescence may extend over a period of three or four days; the fever gradually subsiding, nausea and thirst disappearing, the tongue clearing and appetite returning.

The temporarily-enforced fast and the light diet indicated for a few days after such an attack insures immunity from future trouble for a short time, but as long as the molars are working their way through the gum, an error in diet, or even a return to ordinary food, may occasion a relapse. It will fully repay the physician to inspect the mouth of every six-year old patient (and upward) suffering from acute indigestion, and after making the examination thoroughly, to lance swollen gums over advancing molars, and apply soothing lotions to irritated gums about loosened temporary teeth, or order extraction if admissible. General treatment must be conducted on the same plan as when the attacks, as is often the case, occur independently of dentition. The important points are careful regulation of the diet, or the withholding, in bad cases, of all food for twenty-four hours; counter-irritation to the epigastrium by sinapisms; the use of ice and iced Vichy water to relieve thirst, and the employment of remedies calculated to check nausea, and at the same time act gently on the bowels. A favorite method with the writer is to divide the elements of a Seidlitz powder into equal parts, say eight for a child of six years, dissolve a portion from each in a tablespoonful of cool water, pour together, add cracked ice, and administer while effervescing.

The well-known combination of calomel and soda is also very efficient, for instance:

R. Hydrarg. chloridi mit........................gr. i.
Sodii bicarb...................................gr. vi.
Sacchari........................................gr. xxx.
M. et fr. chart. No. vi.

Sig. One powder every two hours until all are taken or until the bowels act freely.

It is difficult to display two much caution in returning to a full diet, and in spite of the protestations of parents, extreme moderation should be the rule until local irritation be relieved.

Chronic gastro-intestinal catarrh owes its origin to second dentition more frequently than to any other cause save whooping cough. In this condition—so aptly termed by Eustace Smith "Mucous diseases"—there is a mucous flux from the whole internal surface of the alimentary canal which mechanically interferes with the digestion and absorption of food, and greatly impedes nutrition. It may be met with at any time during the eruption of the permanent teeth.

The affected child is emaciated and weak. His face is pale, though subject to marked changes in color. At times a circumscribed crimson flush appears on one or both cheeks. Again, there is so much pallor, particularly about the lips, that fainting seems imminent. The eyes are surrounded by bluish circles that deepen when the face pales; the conjunctivæ are muddy and there is occasional squinting. The skin is sallow, dry and rough; the hair lusterless and faded. There is decided pallor of the oral mucous membrane; the tongue flabby, indented by the teeth, and glazed, and the breath is heavy and fetid. The appetite in the beginning fails, then becomes capricious, and finally is almost insatiable. Eating is followed by a sensation of drowsiness, and by eructations of flatus and acid liquids.

Tympanitic distention of the belly and pain are always present. Pain may be general, when it amounts to little more than a sensation of soreness, or more frequently it is limited to the left hypochondrium, and then is stitch-like in character. Either variety may be constant or present only after meals; in the former case there is a temporary increase of discomfort after eating. Paroxysms of severe pain about the umbilicus, with great pallor of the face, are sometimes observed.

Constipation of the bowels is usual, and the evacuations, which take place at intervals of two or three days are scanty and composed of small, hard, dark-colored lumps, with a quantity of mucus. By day the patient suffers from headache; is languid and ill-tempered. At night he is restless, grinds his teeth, starts from sleep in terror caused by frightful dreams, screams or laughs incoherently, and for a time seems unconscious of his surroundings. Somnambulism and nocturnal incontinence of urine are quite common.

There is no alteration of the temperature, the pulse is feeble, and there is frequently a slight, dry, hacking cough, unquestionably due to reflex irritation of the larynx. The urine is diminished during the continuance of severe pain, but is voided in large quantities at the termination of the paroxysms.

At intervals of two or three weeks violent vomiting and purging occur. During these attacks, which last from one to three days, a large quantity of mucus is expelled; there is slight fever; and the tongue becomes less flabby, more pointed, and covered with a thick, white, feathery fur, except along the sides, where several smooth, red, glazed patches of variable size and shape, with irregularly-indented margins, appear. The cleaning-out process is followed by temporary improvement, but the symp toms slowly return and grow worse to culminate in another attack.

The course of mucous diseases is very protracted, extending over months, and while there is no regular progression the symptoms tend to grow more and more severe as time lapses.

Hacking cough, emaciation and debility may suggest tuberculosis. A diagnosis, however, can be readily made by noting the state of the tongue; the mucous stools; the color and condition of the skin; the absence of pyrexia, except in attacks of vomiting and purging; the periodicity of these attacks; the diurnal drowsiness and nocturnal terrors, and the irregularity of the course. At the same time, the onset of the symptoms, after the commencement of second dentition, is a point of importance.

Mucous disease is not dangerous in itself though by reducing the strength it predisposes to other and much more fatal diseases.

After removing oral irritation so far as possible there are several rules of management to be enforced. First, all articles of diet that readily undergo fermentation must be withheld, and food should be given in moderate quantities and at shorter intervals than in health so that while the stomach is at no time overdistended enough nourishment may be ingested. Of permissible articles milk, eggs, and lean meat are the chief, though fresh fish, raw oysters, cauliflower top, spinach, asparagus, lettuce, and stewed celery can be used without ill effect. With such a list to select from it is an easy matter to write out a suitable dietary, and to make changes sufficiently often to avoid cloying the appetite by monotony. Filtered water or Vichy should constitute the drink, except there be great debility, when whisky or old dry sherry are useful; they must be well diluted and given in carefully-proportioned doses with the meals.

Next, the activity of the skin must be maintained by general baths followed by inunctions of olive oil, and by keeping the surface carefully covered with woollen underclothing. Finally, exercise in the open air on suitable days in winter, and an almost complete outdoor life in summer, greatly favor recovery.

Medical treatment is of minor import but by no means to be neglected. The indications are to check the secretion of mucus; to neutralize the irritating acids formed by fermentation of the food: to restore the mucous membrane to a normal condition, thereby improving secretion, digestion and appetite; and to secure regular action of the bowels and the expulsion of collected mucus and fæces. These ends accomplished, strength and health return, though it may be necessary to call in the aid of tonics.

Alkalies are the best remedies to check the secretion of mucus, and to liquefy it so that it may the more readily be removed. They are also most efficient in neutralizing the acid products of fermentation. Simple bitters, too, have some power in lessening the formation of mucus, and considerable influence in arresting fermentation; at the same time they give tone to the mucous membrane and stimulate digestion. Laxatives keep the bowels clear. Of the first class, bicarbonate of soda; of the second, gentian and columbo; and of the third, senna or aloes are to be preferred.

A very serviceable prescription is—

℞. Potassii iodidi..........................................gr. vi.*

Sodii bicarbonatis...................................ʒ i.

Extract sennæ fld.....................................fʒ iii.

Inf. calumbæ q. s., ad...............................f℥ iii.

M. Sig. Two teaspoonsful three times daily before eating, for a child of six years.

After food it is well to order from ten to twenty drops of tincture of aloes and myrrh in a little water for its powerful tonic action on the intestinal mucous membrane. Should tonics be needed in the after treatment, tincture of nux vomica and

* The iodide of potassium is used to increase the activity of the salivary and gastro-intestinal glands.

ferrated elixir of chinchona are the best. In order to prevent a relapse, mixed diet must be avoided for at least two months after convalescence is fully established, and to confirm the cure a change of air by a trip to the mountains or seashore is advisable.

Diarrhœa is a very constant attendant upon second dentition. It is most apt to arise in the changeable weather of spring and autumn or in the heat of July, August or September. In this respect it resembles the diarrhœa so common with primary dentition, but unlike the latter, it shows little or no tendency to run into entero-colitis. Two forms are met with—namely, catarrhal diarrhœa and lientary. Catarrhal diarrhœa is accompanied by slight furring of the tongue, loss of appetite, and abdominal pain of a colicky nature. The evacuations are frequent, light yellow in color, and oflensive, semi-solid or liquid. For the relief of these cases all that is required, beyond attention to the mouth, is a bland diet, perhaps a dose of castor oil, and some mild astringent mixture. For example, let the patient take for breakfast a soft-boiled egg, milk with lime water, and stale, dry bread; for dinner, some meat broth (free from fat), stale, dry bread, and rice and milk pudding; for supper, milk and stale, dry bread. After the dose of oil, to be given when pain is marked, a good astringent mixture is—

R. Tr. opii. deod.....................................♏ xii.

Bismuth. subnitratis................................ʒ i.

Syrupi...........................................f℥ ss.

Mist. cretæ. q. s., ad...........................f℥ iii.

M. Sig. Two teaspoonsful every two hours.

The treatment may terminate with a course of wine of pepsin, or of pepsin with dilute muriatic acid.

Lientery depends upon great irritability of the intestine and exaggerated peristalsis. The stools are limited to four or five a day and are composed of almost completely undigested food and mucus. An evacuation occurs in the morning soon after rising, the others during or directly after meals. They are preceded by griping pain and by so urgent a desire that the little sufferer has difficulty in waiting for the chamber or reaching the closet. Nux vomica, followed by arsenic, are the remedies to be employed. For instance, until the stools become less frequent and urgent, and the griping pain diminishes, a good prescription is—

R. Tr. opii deod.,
Tr. nucis. vom., aa............................mi. xlviii.
Aq. menth. pip., q. s., ad.......................f℥ iii.

M. Sig. One teaspoonful before each meal, at the age of six years.

Afterwards—

R. Liquor. potassii arsenitis..........................f℈ i.
Inf. gentianæ com., q. s., ad... .................f℥ iii.

M. Sig. One teaspoonful after each meal.

Of course the diet must at the same time be carefully regulated.

The general depression produced by the coming of the second teeth would naturally favor the development of any constitutional tendency, and having traced the connection in several instances, I have no doubt that not a few cases of tubercular ulceration of the bowels owe something to this etiological factor.

*d.* Enlargement of the submaxillary gland, and one or more of the lymphatic glands of the neck, is a frequent occurrence during the approach of the first molors. Patients so affected may, at the same time, present other evidences of difficult dentition; for example, they often show the symptoms of "general debility" already referred to, and often have mucous disease, but they are quite as frequently perfectly healthy in constitution as strumous.

The swelling of the gland or glands is moderate in degree; there is considerable hardness, moderate tenderness and pain, but the super-imposed skin is movable and healthy. There is some tendency to chronic enlargement and induration, though little or none to suppuration, save in scrofulous subjects.

Should the gums be thoroughly lanced—and it is often necessary to sink the knifeblade deep to free the coming tooth—the glandular swelling soon subsides, though resolution can be hastened by painting with tincture of iodine, or using the following ointment:

R. Ichthyol......................................... ℈ i.

Lanolin ...........................................℥ i.

M. Sig. Apply to the enlarged glands three times daily, with rubbing.

*e* and *f*. Conjunctival blennorrhœa and otitis, sometimes noticed during primary dentition, have come within my observation, but from the intimate connection between the nerves of the ear and eye and those of the teeth, it is more than probable that certain other disturbances of these two organs of special sense arise during second dentition and depend upon dental irritation. Unfortunately so few cases of disease of these two organs come under my notice, and so little attention has been paid to this causal relation by specialists, that I have no data upon which to base conclusions.

*g*. Herpes of the lips, eczema, and urticaria frequently appear during the eruption of the second teeth. They apparently depend upon gastro-intestinal disturbances, and are relieved by measures directed to the cure of disorders of this tract, together with appropriate local treatment, and attention to the teeth and gums.

*h*. Nasal catarrh, "teething cough," and an increased susceptibility to catarrh of the bronchial mucous membrane are the chief affections of the respiratory tract.

Nasal catarrh is generally subacute in type, and is attended by hypertrophy and redness of the Schneiderian membrane. There is a more or less copious discharge, which has a slight, heavy odor, may be composed of thin mucus or of muco-pus, and which sometimes dries into thick crusts. This catarrh occasionally runs into ozæna in weak and strumous children.

Attention to the teeth, frequent syringing of the nostrils with insufflation of boracic acid and bismuth (1 part to 2), the occasional application of a weak solution of nitrate of silver (gr. v to f℥ i), judicious use of the electro-cautery, and the administration of tonics constitute the best treatment for the ordinary form of catarrh. Ozæna calls for its special plan of management. "Teething cough" is due to reflex irritation of the pneumogastric nerve; it is identical with the "stomach cough" of mucous disease. Bronchitis can never be said to be due directly to dentition. This cause acts only indirectly by reducing general vitality and the power of resisting disease, and thus rendering the delicate bronchial mucous membrane more than ordinarily susceptible to catarrhal inflammation from chilling of the surface and exposure to damp air. An attack of bronchitis may occur with the eruption of one group of permanent teeth or the attack may be repeated with several successive sets.

I have in my mind now the case of a delicate boy who, in spite of the utmost care on the part of his mother, had a most severe bronchitis during the eruption of the six-year molar, and a second persistent attack while the four central incisors were pushing their way through. Not two months before writing this he again came under my care suffering from a third attack that promised equal obstinacy. On inspecting the mouth the four lateral temporary incisors were found to be loose, and their permanent substitutes evidently advancing rapidly. Extraction of the loose teeth was ordered, together with a mild expectorant and a tonic mixture, and the catarrh soon subsided.

*i.* Nervous disorders both sensory and motor are encountered.

Headache is common. The pain is usually temporal and unilateral. It may be seated, however, in the occipital region, or in different parts of the face, and sometimes shifts suddenly from the temporal to the occipital region. It is lancinating, more or less constant, with no distinct intermissions, and during its continuance there is restlessness, anorexia, a frequent, hard pulse, sweating, dilatation of the pupil on the affected side, and perhaps dimness of vision, diplopia, and colored or uncolored spectra. One or more painful points can often be detected, and generally there is a hard, tender, moderately enlarged lymphatic gland in the submaxillary or cervical region.

These attacks are due to disordered vaso-motor innervation, depending upon irritation of the sympathetic nerves and producing irregular contraction or spasm of the vessels—the temporal or occipital artery, as the case may be. The real source of irritation is to be found in the mouth. The mode of action may be twofold. First, from a swollen gum or carious tooth, the lymphatic vessels readily convey irritating matter to a neighboring lymph gland, and the irritation here excited acts, in its turn, as a disturber of the sympathetic nerves which furnish the vaso-motor supply to the carotid artery and its branches. This theory of the production of migraine has the value of the support of T. Lauder Brunton.

The other method of production—and this I simply submit for consideration—is one of direct nerve connection, the submaxillary ganglion acting in the case of the lower teeth and the spheno-palatine in the upper, as the medium of transfer of irritation to the vaso-motor nerves.

Bromo-caffeine, gelsemium, saline laxatives, and tonics may be employed to lessen the severity of the pain, but lancing, extraction, or proper "filling" are the only certain remedial measures.

The motor disturbances, while not quite as common as the sensory, are more varied. Reflex spasms and paralysis of the eyelid have been noted.* The former I have frequently seen, but the latter, so far as I can recall, has never fallen within my experience. For the reflex spasms no method of treatment availed until the dental irritation was removed, and the same statement may be hazarded *a priori* in regard to paralysis of the lid.

More extended paralysis also occurs. In this connection I can do no better than quote the words of Brunton, the correctness of which I can fully confirm. After speaking of paralysis of the eyelid, this author states: "Sometimes, however, paralysis occurs of a much more extensive character in consequence of dental irritation, especially in children. Teething is recognized by Romberg and Henoch† as a frequent cause of paralysis, appearing in children without any apparent cause.‡ According to Fliess, paralysis of this sort occurs more commonly during the period of the second dentition, whereas convulsions generally occur during the first. Its onset is sudden. The child is apparently in good health, but at night it sleeps restlessly and is a little feverish. Next morning the arm, or more rarely the leg, is paralyzed. The arm drops; it is warm but swollen, and of a redish-blue color. It is quite immovable, but the child suffers little or no pain. Not unfrequently paralysis is preceded by choreic movements. Sometimes recovery is rapid, but at other times the limb atrophies, and the paralysis may become associated with symptoms indicating more extensive disturbance of the spinal cord and brain, such as difficulty of breathing, asthma, palpitation, distortion of the face, and squint, ending in coma and death.

It is only in very rare instances that we are able to gain any insight into the pathological anatomy of such cases, because they rarely prove fatal, and even when they do, the secondary changes are generally so considerble as to leave one in doubt as to the exact mode of commencement. This renders all the more valuable the case recorded by Fliess, in which a boy five years old, and apparently quite healthy, found his left arm completely paralyzed on awakening one morning after a restless night. The arm was red, but the boy suffered no pain, and played about without paying much attention to the arm. The same day he fell from a wagon upon his head and died in a few hours. Apart from the fracture of the skull, which caused his death, the anatomical appearances which were found were congestion of the spinal cord, and great reddening and congestion of the meninges near the point of origin of the brachial nerves, where the veins were also much fuller than on the corresponding right side. There was no organic change perceptible, either in the spinal cord or in the brachial nerves. On the other hand, the turgescence of the veins extended from the shoulder and neck up to the face, and was very striking in the submaxillary region.

This vascular congestion seems to point to vaso-motor disturbance of a somewhat similar kind to that which we have already noticed in connection with occipital headache, or with migraine, accompanied by subjective appearance of either form or color.*

This form of paralysis certainly suggests acute anterior poliomyelitis, though the symptoms are not quite identical, and the age is not that at which "infantile palsey" usually occurs.

Unfortunately, in all but the mildest cases, which get well quickly, when the paralysis appears the mischief is done, and little benefit can be expected from attention to the teeth. The treatment must be conducted on the plan usually adopted in infantile paralysis.

As just stated, dental irritation sometimes produces choreic movements as prodromata of paralysis, but it much oftener acts as the exciting cause of genuine chorea in nervous children. Approaching molars, or carious, loose milk teeth about to be shed, may be the source of irritation. The causal relation is proved by the fact that the chorea disappears or yields quickly to ordinary treatment when the new teeth pierce the gums or are freed by lancing, or when decayed teeth are removed. Epilepsy is another nervous affection which can occasionally be traced to the same source. In such cases one usually finds a history of repeated general convulsions during primary dentition.

1820 S. Rittenhouse Square. *Therapeutic Gazette.*

---

Foolish Use of Carbolic Acid.—Prof. Billroth, of Vienna, warns once more against the imprudent use of carbolic acid, as follows: In the last month four cases have come to my notice where fingers with insignificant wounds have become gangrenous by foolish application of carbolic acid. All those four cases were children whose parents had prescribed a carbolic acid bandage by their own authority, because carbolic acid was said to be good for healing wounds. The use of carbolic acid is much more limited in surgery than heretofore; it is only by degrees we have known the dangers it may present. This remedy may not only cause inflammations and gangrene; it may kill by blood-poisoning. Its good qualities are developed only in the hands of a competent physician. I dissuade, most emphatically, the application of carbolic acid without the prescription of a physician. I recommend as the best bandage for fresh wounds acetate of lead (lead-water), which is for sale in all drug stores.—*Pharmaceutische Post.*

